# Molecular characterization, antibiotic resistance pattern and capsular types of invasive *Streptococcus pneumoniae* isolated from clinical samples in Tehran, Iran

**DOI:** 10.1186/s12866-020-01855-y

**Published:** 2020-06-16

**Authors:** Maryam Beheshti, Fereshteh Jabalameli, Mohammad Mehdi Feizabadi, Farhad Bonakdar Hahsemi, Reza Beigverdi, Mohammad Emaneini

**Affiliations:** Department of Microbiology, School of Medicine, Tehran University of Medical Sciences, Building No. 7, 100 Poursina St., Keshavarz Blvd, Tehran, 14167-53955 Iran

**Keywords:** Invasive *Streptococcus pneumoniae*, Antibiotic resistance, MDR, MLST

## Abstract

**Background:**

*Streptococcus pneumoniae* causes serious infections worldwide. The aim of this study was to determine the molecular characteristic, antibiotic resistance pattern and capsular types of invasive *S. pneumoniae* in Tehran, Iran.

**Results:**

Of the 44 pneumococcal invasive isolates, 39 (89%) were isolated from children and 5 (11%) from adults. The results show that all pneumococcal isolates were susceptible to linezolid but had varying resistance to trimethoprim-sulfamethoxazole (86%), erythromycin (73%), tetracycline (66%), clindamycin (43%), penicillin (16%), chloramphenicol (14%) and levofloxacin (2%). The range of erythromycin, tetracycline and penicillin MICs were 2 - ≥ 256 μg/mL, 4 - ≥ 48 μg/mL, and 0.047 - ≥ 256 respectively. All of the penicillin resistant isolates were multidrug resistant (MDR) and in addition to penicillin were resistant to tetracycline, erythromycin and trimethoprim-sulfamethoxazole. The most common capsular types detected in 64% of the pneumococcal isolates was 6A/B, 19A, 15A, 23F. The multilocus sequence typing (MLST) of 10 pneumococcal isolates revealed 9 different sequence types (STs), including ST 15139 (capsular type 19A) and ST 15140 (capsular type 23F), which have not previously been reported.

**Conclusions:**

The study revealed that the *S. pneumoniae* isolates belonged to diverse capsular types and clones with high rate of resistance to erythromycin, tetracycline, and penicillin.

## Background

*Streptococcus pneumoniae* is the leading cause of invasive disease in young children, older adults and individuals with impaired immune systems [1, 2]. The invasive pneumococcal disease (IPD) is an important cause of morbidity and mortality worldwide [1]. IPD is described by isolation of *S. pneumoniae* from a normally sterile site, such as blood; cerebrospinal fluid (CSF), and pleural or ascitic fluid [1]. The polysaccharide capsule is the main virulence factor in IPD, providing protection the bacterium from the host’s immune system. To date 99 capsular types have been identified based on the antigenic capsular polysaccharide [3]. The introduction of pneumococcal conjugate vaccines (PCV7/10/13) has reduced the incidence of IPD. But, emerging nonvaccine serotypes, commonly 1, 7F, 12F, 15B/C, 22F, 24F, 23B, 33Fand 38 were related to an increase in IPD rates among children and adults [2, 4, 5].

The incredible capacity of *S. pneumoniae* to uptake genes has facilitated the spread of resistance in the pneumococcal population to penicillin and other antibiotics such as macrolides that used routinely to treat the disease [6–9]. The resistance mechanism to penicillin is structural modification in the penicillin binding proteins (PBPs) which have a major role in the synthesis of cell wall. Six PBPs have been identified in *S. pneumoniae* of which three PBPs (*PBP2b*, *PBP2x* and *PBP1a*) are the most often associated with penicillin resistance [9, 10]. While, macrolide resistance mechanisms in *S. pneumoniae* is conferred by two mechanisms. The major resistant determinant is acquisition of the *ermB* gene that encodes a methylase [9, 11, 12]. The second mechanism is acquisition of *mefA/E* genes that encoding an active efflux pump [9, 11, 12]. Noticeably, the majority of isolates that encode *ermB* exhibit the MLS_B_ (Macrolide, lincosamide and streptogramin B) phenotype. While, the majority of isolates that carries *mef* reveal the M phenotype [9, 11–13]. Also the most common mechanism of resistance to tetracycline in *S. pneumoniae* is acquisition one of the two genes, *tetM* and less frequently the *tetO* genes [12, 14, 15] both of which located in mobile genetic elements such as transposons and encode ribosomal protection proteins [14, 15]. Interestingly, resistance to erythromycin and tetracycline is generally related to the insertion of the *ermB* gene into the transposons that contains *tetM* gene, raising worry about the role of tetracycline-resistant strains in the spread of macrolides -resistant strains. The high prevalence of tetracycline resistance among macrolide resistant *S. pneumoniae* has reported [9, 12]. As well, the transposons of the Tn916 or Tn917 family such as Tn6002, Tn3872, Tn6003 and Tn1545 have been described in pneumococci [9, 12]. Also, the main source of the *tetM* gene is Tn916 family [14].

Many molecular methods have been used to determine the genotypic background of *S. pneumoniae*. One of these methods is multilocus sequence typing (MLST), which relies on polymerase chain reaction (PCR) and sequencing of house-keeping genes [2, 6, 16, 17]. The most common sequence types (STs) in Canada were ST320 that is a frequently multidrug resistant (MDR) type and ST695, associated with susceptibility to all antibiotics except for clarithromycin [2]. The most prevalent STs reported in some Asian countries are ST81, ST283 and ST236 [18].

The purpose of the current study was to analyze the molecular characteristic, antibiotic resistance pattern and capsular types of invasive *S. pneumoniae* in Tehran, Iran.

## Results

The pneumococcal isolates were obtained from blood cultures. Of the 44 pneumococcal invasive isolates, 39 (89%) were isolated from children and 5 (11%) from adults.

The antibiotic susceptibility pattern and molecular characteristics of pneumococcal isolates are summarized in Table 1. The results show that all isolates were susceptible to linezolid but had varying resistance to trimethoprim-sulfamethoxazole (86%), erythromycin (73%), tetracycline (66%), clindamycin (43%), penicillin (16%), chloramphenicol (14%) and levofloxacin (2%). The range of erythromycin, tetracycline and penicillin MICs were 2 - ≥ 256 μg/mL, 4 - ≥ 48 μg/mL, and 0.047 - ≥ 256, respectively.

**Table 1:** Antimicrobial resistance pattern, antibiotic resistance genes, virulence genes, capsular type and sequence type of isolates

Isolate	Resistance		MIC (μg/ml)			Tn	Virulence Factor Genes	Capsular type	ST
	Phenotype	genes	P	E	T				
1	**CD, E, Oxa, T, TS**	***ermB, mefA/E, tetM***	**≥256**	**≥256**	**24**	**–**	***cbpA, cpsA, lytA***	***11A***	**9533**
2	**Oxa, TS**	***tetM***	**0.75**	**ND**	**ND**	**–**	***cbpA, cpsA, lytA, ply, pspA***	***–***	**–**
3	**CD, E, Oxa, T, TS**	***ermB, tetM***	**1.5**	**≥256**	**16**	**–**	***cbpA, cpsA, lytA, ply, pspA***	***19A***	**15,139**
4	**Oxa, TS**	***mefA/E***	**0.094**	**ND**	**ND**	**–**	***cbpA, cpsA, lytA, ply, pspA***	***–***	**12,224**
5	**CD, E, Oxa, T, TS**	***ermB, mefA/E, tetM***	**48**	**≥256**	**12**	**–**	***cbpA, cpsA, lytA***	***11A***	**9533**
6	**CD, E, Oxa, T, TS**	***ermB, mefA/E, tetM***	**48**	**≥256**	**16**	**–**	***cbpA, cpsA, lytA, ply***	***15B/C***	**–**
7	**CD, E, Oxa, T, TS**	***ermB, mefA/E, tetM***	**0.19**	**≥256**	**8**	**–**	***cbpA, cpsA, lytA, ply, pspA***	***19A***	**–**
8	**CD, CLR, E, Oxa, T, TS**	***ermB, mefA/E, tetM***	**48**	**≥256**	**16**	**–**	***cbpA, cpsA, lytA, ply***	***23F***	**15,140**
9	**CD, CLR, E, Oxa, T, TS**	***ermB, tetM***	**48**	**≥256**	**16**	**–**	***cbpA, cpsA, lytA, ply, pspA***	***23F***	**–**
10	**CD, CLR, E, Oxa, T, TS**	***ermB, tetM***	**1.5**	**≥256**	**8**	**–**	***cbpA, cpsA, lytA, ply, pspA***	***23F***	**–**
11	**Oxa, T, TS**	***tetM***	**1**	**ND**	**4**	**–**	***cbpA, cpsA, lytA, ply, pspA***	***15A***	
12	**E, Oxa, TS**	***mefA/E***	**0.125**	**8**	**ND**	**–**	***cbpA, cpsA, lytA, ply, pspA***	***14***	**–**
13	**CD, CLR, E, Oxa, T, TS**	***ermB, mefA/E, tetM***	**32**	**≥256**	**24**	**–**	***cbpA, cpsA, lytA, ply, pspA***	***23F***	**–**
14	**E, Oxa, TS**	***mefA/E***	**1**	**12**	**ND**	**–**	***cbpA, cpsA, lytA, ply***	***6A/B***	**–**
15	**Oxa, TS**	**–**	**0.38**	**ND**	**ND**	**–**	***cbpA, cpsA, lytA, ply***	***6A/B***	**–**
16	**CD, E, Lvo, Oxa, T, TS**	***ermB, tetM***	**0.5**	**≥256**	**8**	**–**	***cbpA, cpsA, lytA, ply***	***19A***	**–**
17	**TS**	**–**	**1**	**ND**	**ND**	**–**	***cbpA, cpsA, lytA, ply***	***15A***	**–**
18	**CD, CLR, E, Oxa, T, TS**	***ermB, tetM***	**2**	**≥256**	**12**	**6002**	***cbpA, cpsA, lytA, ply***	***23F***	**–**
19	**E, TS, Oxa**	***mefA/E***	**0.047**	**2**	**ND**	**–**	***cbpA, cpsA, lytA, ply, pspA***	***6A/B***	**–**
20	**E, CD, T, Oxa, TS**	***ermB, tetM***	**0.5**	**≥256**	**12**	**6002**	***cbpA, cpsA, lytA***	***15A***	**–**
21	**E, Oxa, TS**	***ermB, mefA/E***	**0.19**	**3**	**ND**	**–**	***cbpA, cpsA, lytA***	***15B/C***	**1888**
22	**CD, E, Oxa, T, TS**	***ermB, tetM***	**0.5**	**≥256**	**8**	**6002**	***cbpA, cpsA, lytA***	***15A***	**–**
23	**Oxa, TS**	***tetM***	**1.5**	**ND**	**ND**	**–**	***cbpA, cpsA, lytA, pspA***	***15A***	**–**
24	**E, Oxa, T, TS**	***mefA/E, tetM***	**0.5**	**12**	**12**	**2009**	***cbpA, cpsA, lytA, ply***	***9 V***	**–**
25	**Oxa, T**	***tetM***	**1.5**	**ND**	**8**	**916**	***cbpA, cpsA, lytA, pspA***	***1***	**–**
26	**E, Oxa, T, TS,**	***mefA/E, tetM***	**16**	**2**	**24**	**2009**	***cbpA, cpsA, lytA***	***19A***	**1339**
27	**CD, E, Oxa, T, TS**	***ermB, tetM***	**0.38**	**≥256**	**16**	**–**	***cbpA, cpsA, lytA, ply, pspA***	***14***	**–**
28	**Oxa, TS**	***–***	**1**	**ND**	**ND**	**–**	***cbpA, cpsA, lytA, ply***	***6A/B***	**–**
29	**CD, CLR, E, Oxa, T, TS**	***ermB, tetM***	**2**	**≥256**	**24**	**6002**	***cbpA, cpsA, lytA, ply***	***23F***	**–**
30	**CD, E, Oxa, T, TS**	***ermB, tetM***	**1**	**24**	**16**	**–**	***cbpA, cpsA, lytA, ply***	***19F***	**–**
31	**E, Oxa, TS**	***mefA/E***	**0.75**	**3**	**ND**	**–**	***cbpA, cpsA, lytA, ply, pspA***	***6A/B***	**–**
32	**CD, E, T, TS**	***ermB, tetM***	**0.094**	**4**	**8**	**3872**	***cbpA, cpsA, lytA, ply***	***19A***	**12,888**
33	**CD, E, T, TS**	***ermB, tetM***	**0.38**	**12**	**24**	**6002**	***cbpA, cpsA, lytA, ply***	***6A/B***	**–**
34	**E, Oxa, TS**	***mefA/E***	**0.75**	**4**	**ND**	**–**	***cbpA, cpsA, lytA, ply***	***9 V***	**–**
35	**E, Oxa, TS**	***mefA/E***	**0.094**	**4**	**ND**	**–**	***cbpA, cpsA, lytA, ply***	***6A/B***	**–**
36	**Oxa**	***mefA/E***	**0.75**	**ND**	**ND**	**–**	***cbpA, cpsA, lytA***	***19A***	**–**
37	**Oxa, T, TS**	***tetM***	**0.75**	**ND**	**8**	**916**	***cbpA, cpsA, lytA, ply***	***–***	**–**
38	**Oxa, T, TS**	***ermB, tetM***	**0.5**	**ND**	**4**	**6002**	***cbpA, cpsA, lytA, ply***	***15A***	**–**
39	**Oxa, T, TS**	***tetM***	**4**	**ND**	**8**	**916**	***cbpA, cpsA, lytA, ply***	***19A***	**–**
40	**E, Oxa, T**	***ermB, mefA/E, tetM***	**1**	**3**	**16**	**1545/6003 + MEGA**	***cbpA, cpsA, lytA, pspA***	***11A***	**–**
41	**E, Oxa, T**	***ermB, mefA/E, tetM***	**0.75**	**2**	**16**	**1545/6003 + MEGA**	***cbpA, cpsA, lytA, pspA***	***11A***	**–**
42	**E, Oxa, T**	***mefA/E, tetM***	**0.094**	**3**	**48**	**2009**	***cbpA, cpsA, lytA, pspA***	***15A***	**–**
43	**CD, E, Oxa, T, TS**	***ermB, mefA/E, tetM***	**0.75**	**≥256**	**6**	**2010**	***cbpA, cpsA, lytA, ply***	***19F***	**2533**
44	**E, Oxa**	***mefA/E***	**0.19**	**4**	**ND**	**–**	***cbpA, cpsA, lytA, ply, pspA***	***6A/B***	**1876**

*CD* clindamycin, *CLR* chloramphenicol, *E* erythromycin, *Lvo* levofloxacin, *Oxa* oxacillin, *P* penicillin, *T* tetracycline, *TS* trimethoprim/sulfamethoxazole, *ND* non-determined, *Tn* transposon, *ST* sequence type

MIC Breakpoints: Penicillin: S ≤ 2; I = 4; R ≥ 8, Tetracycline: S ≤ 1; I = 2; R ≥ 4, Erythromycin: S ≤ 0.25; I = 0.5; R ≥ 1

The co resistance to erythromycin and clindamycin (the constitutive phenotype) was observed in 43% (19/44) of the isolates and resistance to erythromycin, but not to clindamycin (the M phenotype) was observed in 29.5% (13/44) of the isolates. As to macrolide resistant genes, the most prevalent gene was *ermB* found in 52% (23/44) of the isolates, followed by *mefA/E* found in 50% (22/44) of the isolates. The rate of coexistence of *ermB* and *mefA/E* was 23% (10/44). Analysis of resistance genes illustrated the significantly higher prevalence of *ermB* and *tetM* genes in MDR isolates (*P* = 0.0001).

The co resistance to erythromycin and tetracycline was found in 54.5% (24/44) of the isolates. The rate of *tetM* gene and coexistence of *ermB* and *tetM* were 68% (30/44) and 50% (22/44) respectively. Our result also showed that all of the penicillin resistant isolates were MDR and in addition to penicillin were resistant to tetracycline, erythromycin and trimethoprim-sulfamethoxazole.

In the present study, of 44 isolates 36% (16/44) were positive for transposon genes. The most prevalent transposon genes were Tn6002 14% (6/44) and Tn2009 7% (3/44) followed by Tn1545/6003 4.5% (2/44). No significant correlation was found between the type of transposon and the antibiotic resistance pattern (Table 1).

The pneumococcal isolates belonged to the following capsular types: 6A/B (18%), 19A (16%), 15A (16%), 23F (14%), 11A (9%), 14 (4.5%), 15B/C (4.5%), 19F (4.5%), 9 V (4.5%), 1 (2%) and noticeable (7%).

PCR analysis of virulence genes revealed that 100% of clinical isolates harbored the *cbpA*, *cpsA, lytA* genes, whereas 73% (32/44) the *ply* gene and 41% (18/44) the *pspA* gene.

**Fig. 1:**
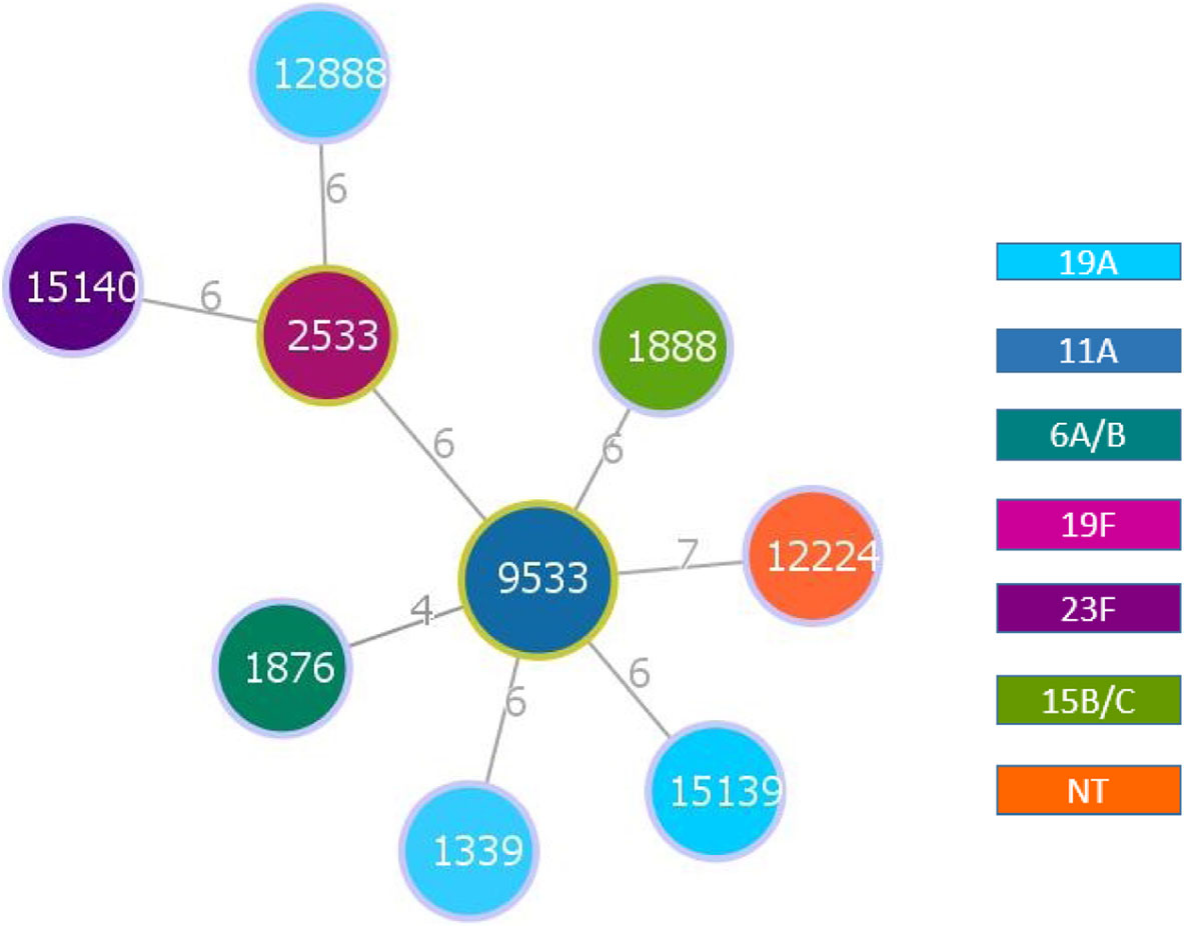
Minimum spanning tree of MLST sequence types of *S. pneumoniae* constructed by PHYLOViZ 2.0. Green outlines indicate a group founder; light blue outlines indicate relatedness to founder; the STs are displayed as circles; Numbers indicate the number of differences between the MLST profiles of the two connected circles; capsular types are characterized by circles different colors

The MLST typing of 10 pneumococcal isolates revealed 9 different ST types, including ST 15139 (capsular type 19A) and ST 15140 (capsular type 23F), which have not previously been reported (Fig. 1). The two ST 9533 isolates belonged to capsular type 11A. Both isolates were resistant to penicillin, erythromycin, clindamycin and tetracycline, but differed in the MIC levels. The MIC of isolate No.1 to penicillin, and tetracycline was > 256 μg/ml and 24 μg/ml, respectively, whilst isolate No.5 was 48 μg/ml and 12 μg/ml, respectively. No significant correlation was found between capsular types and STs (Table 1).

## Discussion

This study investigated molecular characteristics, antimicrobial resistance patterns and capsular types of *S. pneumoniae* isolated from invasive disease in Tehran, Iran. Of the 44 isolates, 73, 68 and 16% were resistant to erythromycin, tetracycline and penicillin, respectively. In a study conducted by Houri et al. in 2017, the percentages of resistance to erythromycin, tetracycline and penicillin in *S. pneumoniae* obtained from two children’s hospitals in Teheran were 71.4, 66.9 and 19.4%, respectively [19]. In another study, conducted by Talebi et al. in 2016, the percentages of resistance to tetracycline and penicillin in the erythromycin resistant *S. pneumoniae* isolates (ERSP) were 85 and 28% [20]. High resistance rates to erythromycin (80.2%), tetracycline (91.2%) and penicillin (47.3%) were reported from China [21]. In our study, the major mechanism conferring resistance to macrolide antibiotics was the constitutive phenotype (43%) mostly correlated with the *ermB* resistance gene (52%). The findings of the current study were in agreement with previous reports from our country, in which *ermB* (50%) was the most frequent genetic determinant among ERSP (20). In a study in Turkey, Kittana et al. reported that the majority of the ERSP isolates (88.2%) had a constitutive phenotype and 89.1 and 50% of the ERSP isolates harbored *ermB* and *mefE* genes, respectively [22]. In contrast to the findings in our study, the M phenotype encoded by *mefA* gene (53%) was more frequently found in Canada [23]. These differences in the prevalence of macrolide resistance and different phenotypes of resistance may be related to the dissemination of multiresistant clones and different patterns in use of macrolides, which led to the variation of resistant phenotypes [24]. In our study, 23% of strains harbored both *ermB* and *mefA* genes, which is in accordance with a report from Turkey (20%) [25]. The majority of macrolide-resistant strains 75% (24/32) were also resistant to tetracycline. This association is due to the insertion of *ermB* into composite transposons of the Tn916 family that contain *tetM* gene [9, 25, 26]. While, the existence of unexpressed *tetM* genes in tetracycline sensitive isolates showed that transposons of the Tn916 family may be more widespread in *S. pneumoniae* than expected to firmly associated with resistant tetracycline [26, 27].

Our study showed that 36% of isolates were positive for transposon genes and among them Tn6002 was more common, which accounts for 14%. Talebi et al. reported that 47% of clinical isolates of *S. pneumonia* were positive for transposon genes and the most predominant transposons were Tn2010 and Tn1545/6003 presented in 29 and 25% of the isolates, respectively [20]. Kittana et al. observed that 88.2% of clinical isolates of *S. pneumonia* harbored transposon genes and Tn2010 (37.2%) and Tn6002 (21.8%) were the most common transposon [22]. Generally, the distribution of pneumococcal transposons and the genes carried by them varies in different parts of the world [22]. One possible explanation for this discrepancy may be explained by the difference in the origin of the isolates as well as other factors.

This study showed that the most common capsular types in the order of frequency were 6A/B, 19A, 15A, 23F, which accounted for 64%. Houri et al. reported that the most common serotypes from blood/CSF were 23F, 19F, 19A and 9 V [19]. Talebi et al. found that serotypes 14 and 19F were the common serotypes isolated from patients with IPD [20]. Habibi Ghahfarokhi et al. observed that the common serotypes isolated from clinical samples were 23F, 14, 3, 19F and 19A [28]. In another study by Azarsa et al. in Iran, the common serotypes isolated from clinical samples were 23F, 19F, 14, 3 and 9 V [29]. In Japan, Sakata et al. evaluated 142 cases of IPD and observed that the most frequently serotypes were 6B, followed by 23F and 19F [30]. In study conducted by Percin et al. in Turkey, the most common invasive serotypes were 1, 19A, 19F, 3, 18C, 6A/B, 14, and 7F [31]. Compared to these reports, almost the same distribution of serotypes responsible for IPD was observed in the current study. The data revealed that serotypes 6A/B, 14, 18C, 19F, 19A, and 23F are the most common pneumococcal serotypes in Asia, particularly in non-vaccine areas [19].

Vaccination has been shown to be effective in reducing the rates of IPD associated with multiple drug resistance [4, 5]. But, vaccination against the most common serotypes of *S. pneumoniae* using PCVs is not still included to the routine immunization program in the Iran and only recommended for high-risk groups [28]. The present study, in addition to others from Iran, shows that the PCV13 could cover the majority of the invasive pneumococcal isolates [19, 20, 28, 29].

Capsular type 19A is frequent among MDR isolates which has already been described in many non-vaccinated regions such as Korea [32]. Researchers have formerly exhibited that the spread of MDR capsular types 19A isolates is due to antibiotic misuse in developing countries [33]. In our country, irrational use of antibiotics has contributed to the emergence of MDR isolates.

In current study, more than 70% (5/7) of capsular type 19A isolates were MDR and showed resistance to erythromycin (majority MIC≥256 μg/ mL), tetracycline (MIC ≥8 μg/ mL), clindamycin, and trimethoprim-sulfamethoxazole. One isolate of capsular type 19A in addition of mention antibiotics showed relatively high resistance to penicillin which carried Tn2009. As for, all (6/6) of capsular type 23F isolates and 50% (2/4) of capsular type 11A, 15B/C (1/2) isolates were MDR (high level MIC for erythromycin and tetracycline) as capsular type 19A isolates but 50% of 11A, 15B/C and 23F were resistance penicillin (MIC ≥32–48 μg/ mL).

Despite the importance of capsular type as an invasive determinant, other virulence determinants were also associated with invasive isolates [34]. As our results, the majority of the isolates contained the virulence determinants that probably indicate the essential of virulence determinants in the ability of an isolate to cause invasive disease [34]. Interestingly, *pspA* gene was encoded by the most pneumococcal isolates which only detected in 41% of our isolates. This is in accordance with the study suggesting that probable limitation of detection by conventional PCR and confirmed this hypothesis by a quantitative PCR assay at high level detection [35].

As for capsular type is assumed to be more important than genotype in the ability of an isolate led to invasive disease but also underline the role of genetic background in invasion [34, 36, 37]. Since pneumococcal isolates with diverse MLST profiles have showed various pathogenicity potential [36]. According to the other studies suggesting high capsular type and genetic diversities in IPD isolates [12, 37–39], there was important diversity among our isolates base on capsular types and different MLST profiles. In pneumococcal isolates, one of the important factor to selective pressure is use of antibiotics [39, 40], so the antibiotic selection pressure may be led to different genetic diversity of IPD isolates that observed in this study [39, 40]. However, causing agents associated with genetic diversity should be further studied.

Reliable and comprehensive data regarding antimicrobial resistance and genetic characteristics, *S. pneumoniae* are scarce, in Iran. This prompted our research. There were limitations to this study. One major bias is the low number of isolates analyzed, which led to no association was observed between the serotypes/transposons or serotypes/STs and STs/ antibiotic resistant phenotype

## Conclusion

The study revealed that the *S. pneumoniae* isolates belonged to diverse capsular types and clones with high rate of resistance to erythromycin, tetracycline, and penicillin.

## Methods

### Bacterial isolates

A total of 44 invasive pneumococcal isolates were collected from hospitalized patients (inpatients) from 2 teaching hospitals (Imam Khomeini and Tehran Children’s Medical Center) affiliated with the Tehran University of Medical Sciences (TUMS), between October 2016 to September 2017. Only one isolate was investigated per patient. The organisms were identified to the species level using standard biochemical methods based on typical colony morphology, Gram staining, catalase, hemolysis, and optochin sensitivity testing (Difco, USA). To confirm the identification of the isolate as *S. pneumoniae* the *lytA* and *ply* genes were amplified by a PCR, using primers: *LytA-F*, 5′-CGGACTACCGCCTTTATATCG-3′; *lytA*-R, 5′-GTTTCAATCGTCAAGCCGTT-3′ [41] and *ply*-F, 5′-ATTTCTGTAACAGCTACCAACGA-3′; *ply*-R, 5′- GAATTCCCTGTCTTTTCAAAGTC-3′ [42].

### Antibiotic susceptibility determination

Antimicrobial susceptibility testing was performed according to the Clinical Laboratory and Standards Institute (CLSI (guidelines. Disk agar diffusion (DAD) method was performed on Mueller-Hinton agar with 5% defibrinated sheep blood, incubated at 35 °C and 5% CO2 for 20–24 h, and zones of inhibition measured after incubation. All isolates were tested against Oxacillin (1 μg), tetracycline (30 μg), erythromycin (15 μg), levofloxacin (5 μg), chloramphenicol (30 μg), linezolid (30 μg), clindamycin (2 μg), trimethoprim/ sulfamethoxazole (1.25/23.75 μg). All of the antibiotic discs were purchased from Mast Diagnostics Ltd. (Merseyside, UK). Minimum inhibitory concentration (MIC) for erythromycin, tetracycline and penicillin were determined with E-test (0.016–256 μg/ml-Liofilchem, Via Scozia, Italy). The MIC was interpreted according to the CLSI breakpoints [43]. MDR was considered as resistance to three or more different classes of antimicrobial. *S. pneumoniae* ATCC 49619 was used for quality control strain to ensure the reliability of the results.

### Capsular typing

The PCR were performed with capsular specific primers as described by Ahn et al. as Table 2 [44]. First of all the confirmed pneumococcal isolates were examined for amplification of *cpsA gene* (Table 3) [45]. Then, the capsular primers used to detect most common capsular types that were outlined in Table 2 [44].

**Table 2:** Sequences of capsular primers

Primer	Sequence	Amplicon size (bp)	Reference
23 F	5′-GTAACAGTTGCTGTAGAGGGAATTGGCTTTTC-3′ 5′-CACAACACCTAACACACGATGGCTATATGATTC-3’	384	[44]
19F	5’-GTTAAGATTGCTGATCGATTAATTGATATCC-3′ 5′-GTAATATGTCTTTAGGGCGTTTATGGCGATAG-3’	304	
4	5’-CTGTTACTTGTTCTGGACTCTCGATAATTGG-3′ 5′-GCCCACTCCTGTTAAAATCCTACCCGCATTG-3’	430	
6A/B	5’-AATTTGTATTTTATTCATGCCTATATCTGG-3′ 5′-TTAGCGGAGATAATTTAAAATGATGACTA-3’	250	
*14*	5’-CTTGGCGCAGGTGTCAGAATTCCCTCTAC-3′ 5′-GCCAAAATACTGACAAAGCTAGAATATAGCC-3’	208	
*19A*	5’-GTTAGTCCTGTTTTAGATTTATTTGGTGATGT-3′ 5′-GAGCAGTCAATAAGATGAGACGATAGTTAG-3’	478	
*3*	5’-ATGGTGTGATTTCTCCTAGATTGGAAAGTAG-3′ 5′-CTTCTCCAATTGCTTACCAAGTGCAATAACG-3’	371	
*15A*	5’-ATTAGTACAGCTGCTGGAATATCTCTTC-3′ 5′-GATCTAGTGAACGTACTATTCCAAAC-3’	434	
*15B/C*	5’-TTGGAATTTTTTAATTAGTGGCTTACCTA-3′ 5′-CATCCGCTTATTAATTGAAGTAATCTGAACC-3’	496	
*1*	5’-CTCTATAGAATGGAGTATATAAACTATGGTTA-3′ 5′-CCAAAGAAAATACTAACATTATCACAATATTGGC-3’	280	
*11A*	5’-GGACATGTTCAGGTGATTTCCCAATATAGTG-3′ 5′-GATTATGAGTGTAATTTATTCCAACTTCTCCC-3’	463	
*9 V*	5’-CTTCGTTAGTTAAAATTCTAAATTTTTCTAAG-3′ 5′-GTCCCAATACCAGTCCTTGCAACACAAG-3’	753	
*7F*	5’-CCTACGGGAGGATATAAAATTATTTTTGAG-3′ 5′-CAAATACACCACTATAGGCTGTTGAGACTAAC-3’	826

**Table 3:** Sequences of oligonucleotide primers

Primer	Sequence	Amplicon size (bp)	Reference
*lytA*	5’-CGGACTACCGCCTTTATATCG-3′ 5′-GTTTCAATCGTCAAGCCGTT-3’	229	[41]
*ply*	5’-ATTTCTGTAACAGCTACCAACGA-3′ 5′-GAATTCCCTGTCTTTTCAAAGTC-3’	347	[42]
*cpsA*	5’- AGTGGTAACTGCGTTAGTCC − 3′ 5′- CTGCCAAGTAAGACGAACTC − 3’	362	[45]
*erm(B)*	5’-TGGTATTCCAAATGCGTAATG-3′ 5′-CTGTGGTATGGCGGGTAAGT-3’	745	[46]
*mef*(*A/E*)	5’-AGTATCATTAATCACTAGTGC-3′ 5′-TTCTTCTGGTACTAAAAGTGG-3’	346	[47]
*tetO*	5’-AACTTAGGCATTCTGGCTCAC-3′ 5′-TCCCACTGTTCCATATCGTCA-3’	515	[13]
*tetL*	5’-ATAAATTGTTTCGGGTCGGTAAT-3′ 5′-AACCAGCCAACTAATGACAATGAT-3’	1077	[48]
*tetK*	5’-GTAGCGACAATAGGTAATAGT-3′ 5′-GTAGTGACAATAAACCTCCTA-3’	361	[49]
*tetM*	5’-AGTGGAGCGATTACAGAA-3′ 5′-CATATGTCCTGGCGTGTCTA-3’	159	[49]
*aphA3*	5’-GCCGATGTGGATTGCGAAAA-3′ 5′-GCTTGATCCCCAGTAAGTCA-3’	292	[13]
*int*	5’-GCGTGATTGTATCTCACT-3′ 5′-GACGCTCCTGTTGCTTCT-3’	1046	[48]
*xis*	5’-AAGCAGACTGAGATTCCTA-3′ 5′-GCGTCCAATGTATCTATAA-3’	194	[48]
*tnpR*	5’-CCAAGGAGCTAAAGAGGTCCC-3′ 5′-GTCCCGAGTCCCATGGAAGC-3’	1548	[48]
*tnpA*	5’-GCTTCCATGGGACTCGGGAC-3′ 5′-GCTCCCAATTAATAGGAGA-3’	2134	[48]
*tndX*	5’-ATGATGGGTTGGACAAAGA-3′ 5′-CTTTGCTCGATAGGCTCTA-3’	611	[48]
*pspA*	5’-CATAGACTAGAACAAGAGCTCAAA-3′ 5′-CTA CAT TAT TGT TTT CTT CAG CAG-3’	214	[36]
*cbpA*	5’-GCTAATGTAGCGACTTCAGATCAA-3′ 5′-AGCTTGGAAGAGTTTCTTCACCTA-3’	142	[36]

### Detection of resistance genes, virulence genes and transposon profiles

The PCR assays were used to detect the macrolide resistance genes (*ermB* and *mef A/E*), tetracycline resistance genes (*tetM*, *tetO*, *tetL* and *tetK*), virulence genes including autolysin A (*lytA*), pneumolysin (*ply*), pneumococcal surface protein A (*pspA*), and Choline binding protein A (*cbpA)* with primers specific for each gene.

The transposons were detected using PCR assay for Tn916 and Tn917 transposon-related genes including *xis, int, tndX, tnpR* and *tnpA*. The resistance genes related to the different transposons were Tn2009 (*tetM, int, xis, mef*), Tn6002 (*ermB, tetM, int, xis*), Tn3872 (*ermB, tetM, tnpA, tnpR*), Tn2010 (*ermB, tetM, int, xis, mef*), Tn6003/Tn1545 (*ermB, tetM, int, xis, aph3’-III*), Tn6002+ MEGA (macrolide efflux genetic assembly) [12]. All primers are listed in Table 3.

### MLST analysis

MLST was performed with selected isolates using the internal fragments of seven housekeeping gene including *aroE*, *gdh*, *gki*, *recP*, *spi*, *xpt*, and *ddl* as specified by Enright et al. [17].

The sequences types (STs) were determined by the comparison with those of corresponding allelic profiles at MLST database (http://pubmlst.org/spneumoniae/). Minimum spanning trees were produced using PHYLOViZ 2.0 software [50].

### Statistical analysis

The statistical analysis of the difference in the frequency of the pneumococcal genes was evaluated by using the chi-square and Fisher’s as appropriate. The differences less than 0.05 were considered significant statistically.

## Data Availability

All documents and additional data are available from the corresponding author upon reasonable request.

## Abbreviations

IPD: Invasive pneumococcal disease
CSF: Cerebrospinal fluid
MLS_B_: Macrolide, lincosamide and streptogramin B
MLST: Multilocus sequence typing
MDR: Multidrug resistant
MIC: Minimum Inhibitory Concentration
PBPs: Penicillin binding proteins
PCR: Polymerase chain reaction
*lytA*: Autolysin A
*ply*: Pneumolysin
*pspA*: Pneumococcal surface protein A
*cbpA*: Choline binding protein A
MEGA: Macrolide efflux genetic assembly
ST: Sequences type
CLSI: Clinical Laboratory and Standards Institute
DAD: Disk agar diffusion
CD: Clindamycin
CLR: Chloramphenicol
E: Erythromycin (E)
Lvo: Levofloxacin
Oxa: Oxacillin
P: Penicillin
T: Tetracycline
TS: Trimethoprim/sulfamethoxazole
ND: Non-determined
Tn: Transposon
PCV: Pneumococcal conjugated vaccine
ERSP: Eythromycin resistant *S. pneumoniae*
